# Spatiotemporal three-dimensional transport dynamics of endocytic cargos and their physical regulations in cells

**DOI:** 10.1016/j.isci.2022.104210

**Published:** 2022-04-06

**Authors:** Chao Jiang, Mingcheng Yang, Wei Li, Shuo-Xing Dou, Peng-Ye Wang, Hui Li

**Affiliations:** 1 Beijing National Laboratory for Condensed Matter Physics and Laboratory of Soft Matter Physics, Institute of Physics, Chinese Academy of Sciences, Beijing 100190, China; 2 School of Systems Science and Institute of Nonequilibrium Systems, Beijing Normal University, Beijing 100875, China; 3 School of Physical Sciences, University of Chinese Academy of Sciences, Beijing 100049, China; 4 Songshan Lake Materials Laboratory, Dongguan, Guangdong 523808, China

**Keywords:** Optical imaging, Cell biology, Biophysics

## Abstract

Intracellular transport, regulated by complex cytoarchitectures and active driving forces, is crucial for biomolecule translocations and relates to many cellular functions. Despite extensive knowledge obtained from two-dimensional (2D) experiments, the real three-dimensional (3D) spatiotemporal characteristics of intracellular transport is still unclear. With 3D single-particle tracking, we comprehensively studied the transport dynamics of endocytic cargos. With varying timescale, the intracellular transport changes from thermal-dominated 3D-constrained motion to active-dominated quasi-2D motion. Spatially, the lateral motion is heterogeneous with peripheral regions being faster than perinuclear regions, while the axial motion is homogeneous across the cells. We further confirmed that such anisotropy and heterogeneity of vesicle transport result from actively directed motion on microtubules. Strikingly, inside the vesicles, we observed endocytic nanoparticles make diffusive motions on their inner membranes when microtubules are absent, suggesting endocytic cargos are normally localized at the inner vesicle membranes through a physical connection to the microtubules outside during transport.

## Highlights

• Endocytic transport changes from 3D-constrained to quasi-2D motions with timescales
• Lateral motion is heterogeneous while axial motion is homogeneous across the cells
• Microtubules cause the anisotropy and heterogeneity of vesicle transport
• Endocytic particles make diffusive motion on the inner membrane of vesicles

## Graphical abstract

**Figure:**
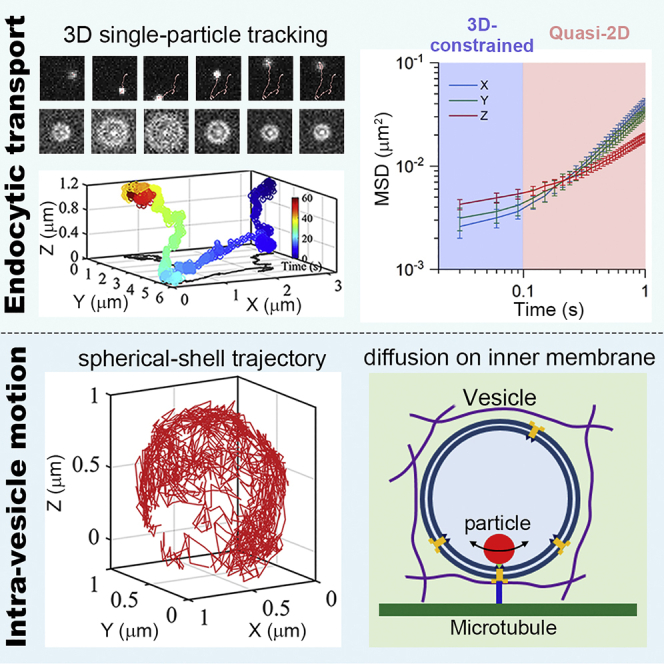

## Introduction

Intracellular transport in cells is the critical way to deliver various biomolecules to maintain the normal cell physiology (Brangwynne et al., 2009; Kamal and Goldstein, 2000; Li et al., 2012, 2018a; Zhang et al., 2021; Yuan et al., 2022; Zajac et al., 2013). Defects in intracellular transport are related to developmental, neurodegenerative, and immunological diseases (Bergman et al., 2018; Chen et al., 2021; Fujii et al., 2021; Tzeng and Wang, 2016; Van Battum et al., 2015; Yarwood et al., 2020). Different from liquid environment, the transport in living cells is driven by both the thermal fluctuations and active forces (Fakhri et al., 2014; Guo et al., 2014; Li et al., 2018a). Besides, the cytoarchitectures as well as molecule crowding also impose multiple regulations to the intracellular transport (Nettesheim et al., 2020; Shen et al., 2021; Wong et al., 2004). As such, the intracellular transport dynamics is far from equilibrium and is complex (Brangwynne et al., 2009; Chen, 2020; Fakhri et al., 2014; Guo et al., 2014).

Previous studies based on two-dimensional (2D) imaging focused on the lateral dynamics of endocytic transport (Grady et al., 2017; Li et al., 2012, 2018a; Witzel et al., 2019; Zajac et al., 2013). In contrast, the axial dynamics is rarely addressed although it is indispensable for the intracellular transport in three-dimensional (3D) cells. For example, axial motions are found to help the vesicle to overcome obstacles or cross the intersection of microtubules (Bergman et al., 2018; Verdeny-Vilanova et al., 2017). And it is reported that the diffusion of quantum dots is not isotropic in adherent cells, with the axial motion being constrained compared with the lateral direction (Jiang et al., 2020). Yet compared with the nano-sized quantum dots which diffuse fast in the cytoplasm, the micron-sized vesicles are tightly surrounded by the actin meshwork (Brangwynne et al., 2009), of which the constraint and the isotropic property are still unclear. Moreover, because each power stroke of motor proteins bursts in milliseconds, such active forces would bring a time-dependent influence on the intracellular transport (Fakhri et al., 2014; Gnesotto et al., 2018; Guo et al., 2014). The physical characteristics of the transport dynamics is thus correlated with the timescales. Therefore, characterizing the 3D vesicle transport in high spatial and temporal resolutions is essential for comprehensively describing the intracellular transport dynamics, and for understanding its regulatory mechanism.

In addition to the vesicle transport, the movement behaviors of endocytic cargos inside a vesicle are unknown. Previous studies have detected the orientation changes of endocytic gold nanorods (Cheng et al., 2021; Kaplan et al., 2018; Sun et al., 2019; Xu et al., 2021); however, they cannot distinguish the rotations of endocytic vesicles from the movements of nanorods inside the vesicles. Without a precise axial location, one cannot elucidate the cargo motion inside the vesicles. Currently, the single-particle tracking (SPT) technique has become widely used to reveal the transport dynamics of individual molecules or particles (Lidke and Wilson, 2009; Metzler et al., 2014; Saxton and Jacobson, 1997). Based on commonly 2D SPT, several methods can be exploited to measure the axial positions of particles, such as active feedback (Hou et al., 2019; Wells et al., 2010), multifocal imaging (Chen et al., 2021; Ram et al., 2008; Shen et al., 2017; Toprak et al., 2007; von Diezmann et al., 2017), and astigmatic 3D detection (Huang et al., 2008; Shen et al., 2017; von Diezmann et al., 2017), enabling us to probe the complex intracellular transport dynamics (Huang et al., 2017; Katayama et al., 2009; Pavani et al., 2009; Shen et al., 2017; Sun et al., 2009; Thompson et al., 2010; Verdeny-Vilanova et al., 2017; von Diezmann et al., 2017; Zhou et al., 2019).

In this work, using the 3D SPT microscopy equipped with two-focal imaging and focus-locking apparatus (Jiang et al., 2020; Toprak et al., 2007), we studied the spatiotemporal transport dynamics of endocytic nanoparticles in cells. We observed that 3D intracellular vesicle transport exhibits different dynamical regimes at short and long timescales, changing from the thermal-dominated 3D-constrained motion when the timescale is shorter than 0.1 s, to the active-dominated quasi-2D motion when the timescale is over 0.1 s. Spatially, the lateral motion shows to be heterogeneous with the peripheral regions faster than the perinucleus, while the axial motion is homogeneous across the cell. We further confirmed that the microtubule-based directed motion leads to a long-time quasi-2D and laterally heterogeneous transport of endocytic vesicles, whereas the actin filaments impose an isotropic constraint on the vesicles. Interestingly, we for the first time observed and quantified the nanoparticle diffusion on the inner membrane of vesicles in cells without microtubules, implying the existence of a physical connection between microtubules and endocytic cargos, which crucially influences the dynamics of the endocytic cargos.

## Results

### Short-time 3D-constrained motion and long-time quasi-2D transport of endocytic vesicles

**Figure 1:**
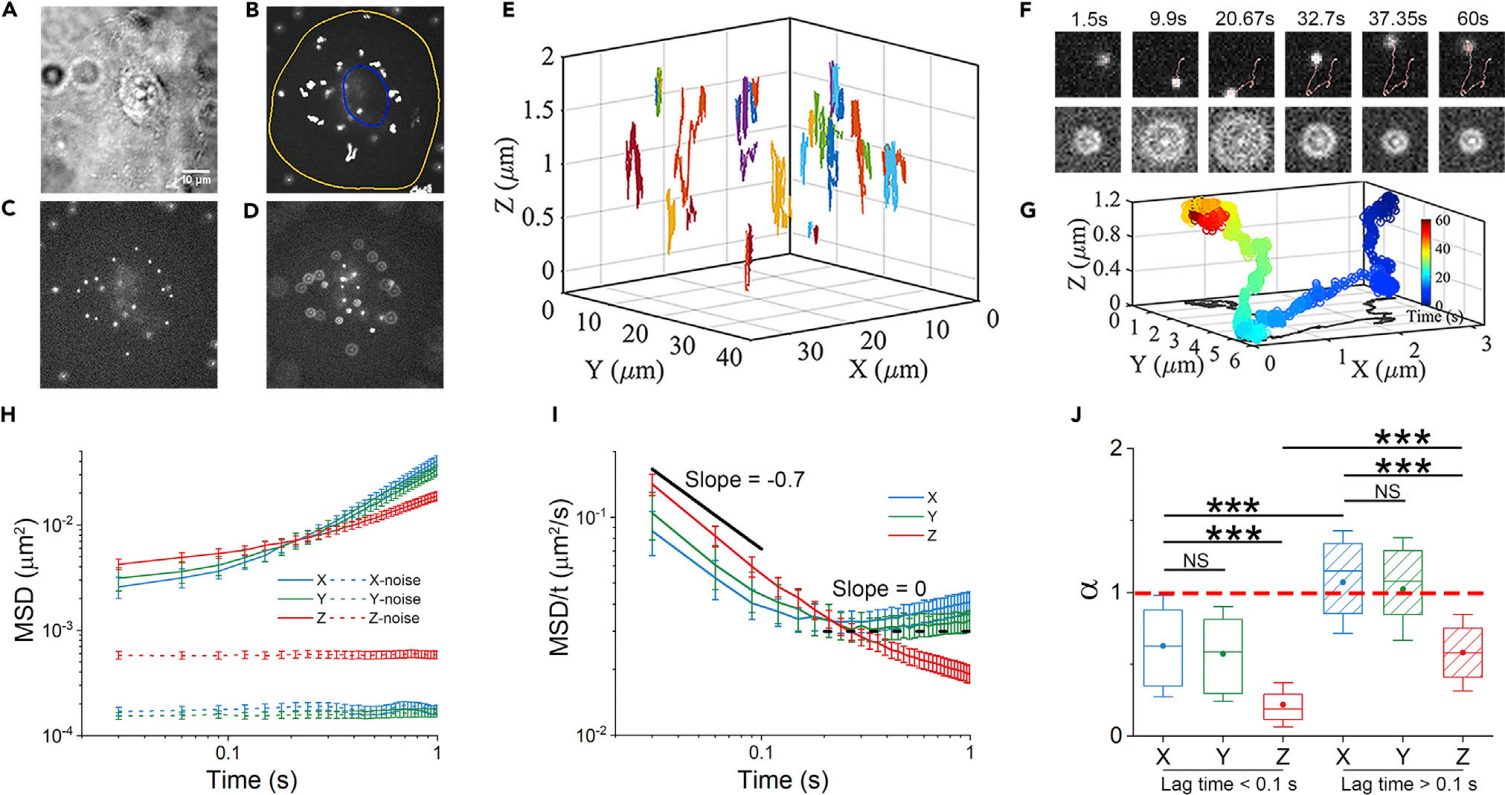
Dynamic of 3D intracellular vesicle transport at different timescales (A) The bright-field image of an A549 cell. Scale bar, 10 μm. (B) Max-projection of fluorescence particle movies. The cell boundary is marked in yellow. (C and D) In-focused (C) and off-focused (D) images of particles in the cell. (E) 3D plots of trajectories (over 50 frames) in different colors within the cell. (F) Time series of a particle in the focused (upper) and off-focused (below) channels, with the trajectory marked. (G) Plot of the trajectory with the time indicated by color. (H) Comparison of the particle MSD in each direction as a function of lag time, in A549 cells (solid lines, 128 trajectories from seven cells) and on the cover glass (noise floor, dash lines, 15 trajectories). (I) Plot of MSD/t versus the time. The solid and dashed line in black color represent the slope of −0.7 and 0, respectively. Error bars indicate the SEM. (J) Comparison of the exponent α in each direction at short and long timescales. For boxplots, the line in the box means median value, the dot means mean value, box edges correspond to 25% and 75% of dataset, error bars indicate SD. ∗∗∗p < 0.001; NS, not significant.

To monitor the intracellular transport, we incubated A549 (human lung carcinoma) cells with 100-nm fluorescent particles for 4 h prior to imaging. These particles underwent internalization through the cell membrane and then endocytic trafficking (Dupont et al., 2013; Gupta et al., 2019). After removing the excess particles, we performed 3D SPT imaging of the intracellular probes in living cells, at 33 Hz frame rate for 1 min (STAR Methods, Figures 1A–1D). The lateral direction (*x*, *y*) is parallel to the coverglass and the axial direction (*z*) is perpendicular to the substrate. The particle axial coordination is measured through the linearly calibrated relationship between the diffraction ring radius and the axial position for immobilized particles on glass (Figures S1A and S1B). The 3D localization accuracy is determined to be lateral 10 nm and axial 17 nm from MSD = 2σ^2^ in each direction (Figure S1C), where σ is the localization accuracy (Jiang et al., 2020; Thompson et al., 2010). Note that we further compared the calibration curves from between the particles in fixed cells and those on glass, and found the similar slopes, indicating that the different refractive indexes between the cell and water have no obvious influence on the measurements of axial movements in our experiments (Figure S2). As the fluorescence particles are enclosed in endocytic vesicles, the particle motions indicate vesicle transports in the cytoplasm. We found that the 3D trajectories provide a more comprehensive picture of transport dynamics within the 3D intracellular environment (Figure 1E). In addition to the typical 2D trajectory of an endocytic particle with laterally directed and diffusive movements, the diffraction ring enlarges and shrinks dramatically (Figures 1F and 1G), revealing that the particle has axial movements simultaneously.

By analyzing 128 trajectories in 7 cells, we plotted the time- and ensemble-averaged mean square displacement (MSD), <Δ*r*(τ)^2^> with Δ*r*(τ) = *r*(*t*+τ) - *r*(*t*) Δr(τ)=r(t+τ)−r(t), and found the anomalous diffusion for endocytic transport (Figure 1H). Note that these MSD data are over ten times larger than the noise floor for fixed particles on the coverglass. With the plot of MSD/t (Figure 1I), we observed two regimes of transport dynamics: at short timescales (*t* ≤ 0.1s), the negative slope of MSD/t indicates the highly sub-diffusive motion, while at long timescales (*t* ≥ 0.1s), the increased slope suggests that the transport gradually alters to normal diffusive motion. To further compare the intracellular transport dynamics in different directions, we used the exponent *α* determined from the nonlinear fitting of MSD with the equation MSD *= A∗t*^α^, as the α values are good indicators of anomalous diffusion. In Figure 1J, the results of α show that at short timescales, the vesicle movements in the three directions are all constrained with the α values being obviously smaller than 1. However, at long timescales, the axial motion remains constrained, while the lateral motion changes from constrained to normal-diffusive and even slightly super-diffusive motion. Furthermore, at long timescales, we estimated the diffusion coefficients by fitting MSD with the equation MSD = 2∗*D*∗*t*, the approximation of which is acceptable as the α values are closer to 1 at the large lag times. It shows that endocytic vesicles diffuse faster in the lateral direction than that in the axial direction (Figure S3A), consistent with the results of the exponent α. Together, these results illustrate that intracellular transport changes from 3D-constrained motion at short timescales to laterally quasi-2D transport at long timescales. We should note that our results do not depend sensitively on the choice of crossover time between short and long timescales (Figure S4). The lateral motion was previously shown by 2D studies (Guo et al., 2014; Gupta and Guo, 2017); we indeed confirm its time-dependent property. More importantly, with the 3D SPT, we found here that the axial motion (*z* direction) of the endocytic particles shows different behaviors from the lateral motion, in which the axial motion is independent on the timescales.

To check whether our 3D tracking with the z-range up to 2 μm would bring biases in the axial measurement, we performed a control experiment by tracking the fluorescent particles in 20% dextran solution (Figure S5). Although the particles diffuse faster than those in cells which is more difficult for tracking, the diffusion was measured to be 3D isotropic, as expected. This confirms that constrained motion in axial direction is resulted from the intracellular environment.

It should be noticed that at short timescales, the exponent *α* in axial direction is smaller than that in lateral direction, suggesting the axial constraint on intracellular endocytic vesicles is stronger, probably due to the laterally distributing cytoarchitectures within adherent cells (Valm et al., 2017). From the increased exponent α of MSD at long timescales, it is inferred the actively directed motion takes places in the lateral direction, but not in the axial direction, which results in the quasi-2D transport. We further demonstrated that the directed motion mainly contributes to the lateral transport, in which the instant velocity in the lateral direction is five times faster than that in the axial direction (Figure S6). The quasi-2D transport of endocytic vesicles together with the quasi-2D diffusion of single QDs we found before (Jiang et al., 2020), suggest that such quasi-2D motion is general for both micron-sized vesicles and nanometer-sized small particles at long timescales.

### Spatial heterogeneity of 3D vesicle transport

**Figure 2:**
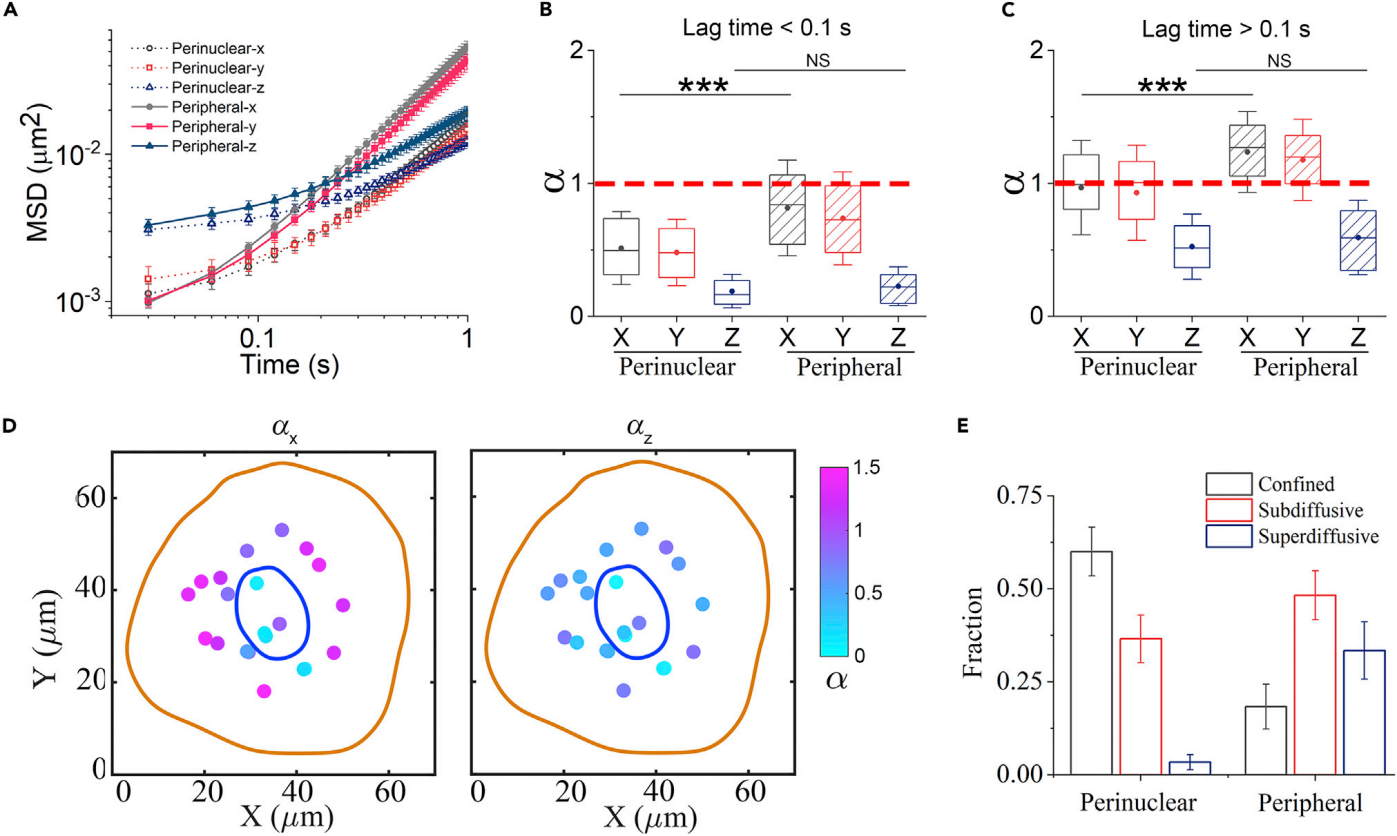
Intracellular transport dynamics in perinuclear and peripheral regions (A) Comparison of MSD for each direction between the perinuclear and peripheral regions. (B and C) Exponent α determined at short timescales (B) and long timescales (C). For boxplots, the line in the box means median value, the dot means mean value, box edges correspond to 25% and 75% of dataset, error bars indicate SD ∗∗∗p < 0.001; NS, not significant. (D) Scatterplots of exponent α in a cell at long timescales for lateral *x* and axial *z* directions, respectively. The values are indicated by the colors. (E) Fraction of confined (α < 0.5), sub-diffusive (0.5<α < 1), and super-diffusive (1<α < 2) motions in the two different regions. 84 trajectories in perinuclear region and 116 trajectories in peripheral region from 14 cells are used. Error bars indicate the SEM.

To further investigate the spatial heterogeneity of intracellular transport, we compared the transport dynamics between the perinuclear and peripheral regions. We defined the circle with 10-μm radius centered at the nucleus as the perinuclear region, and the other area as the peripheral region. The MSD plots show that the vesicle transports in the perinuclear region are obviously slower than those in the peripheral region, at both short and long timescales (Figure 2A). Furthermore, by analyzing the exponent α in each direction (Figures 2B and 2C), we found that the lateral motion in the peripheral region is significantly enhanced when compared with the perinuclear region; however, the axial motion is similarly constrained in both regions. It indicates that the spatial heterogeneity of 3D vesicle transport mainly results from the lateral motion. Moreover, comparison of α maps between the lateral and axial directions clearly illustrates the laterally heterogeneous and axially homogeneous characteristics of 3D intracellular transport dynamics (Figure 2D).

Next, we try to understand the above spatial characteristic of vesicle transport by considering different timescales. At short timescales, the vesicle motion is dominated solely by thermal fluctuations, thus we could access the local cytoplasmic environment that directly influences vesicle motion. The smaller values of exponent α in the perinuclear region suggest that the perinucleus is more crowded than the periphery (Figure 2B). This is consistent with the facts that more organelles are compacted near the cell nucleus (Li et al., 2015) and there is a dense microtubule network in perinuclear region due to vicinity of microtubule-organizing center (Lüders and Stearns, 2007). At long timescale, active forces and directed motion are dominant. From the results of slowly axial motion in both perinuclear and peripheral regions, it can be known that the physical constraint in the axial direction is strong and ubiquitous throughout the cytoplasm (Figures 2C and 2D). Moreover, the lateral motion is remarkably enhanced in the peripheral region compared with the perinuclear region. This suggests that the actively directed transports mainly occur at the peripheral areas of a cell. To further quantify the transport dynamics heterogeneity, we divided the trajectories into confined, sub-diffusive, and super-diffusive motions, based on their exponents α of MSD (Li et al., 2015, 2018a). As expected, the peripheral vesicles show obviously more super-diffusive motions (Figure 2E), consistent with the picture that most directed motions take place at peripheral areas, which leads to the spatial heterogeneity of vesicle transport dynamics.

### Directed motion on microtubules leads to quasi-2D and heterogeneous transport dynamics

**Figure 3:**
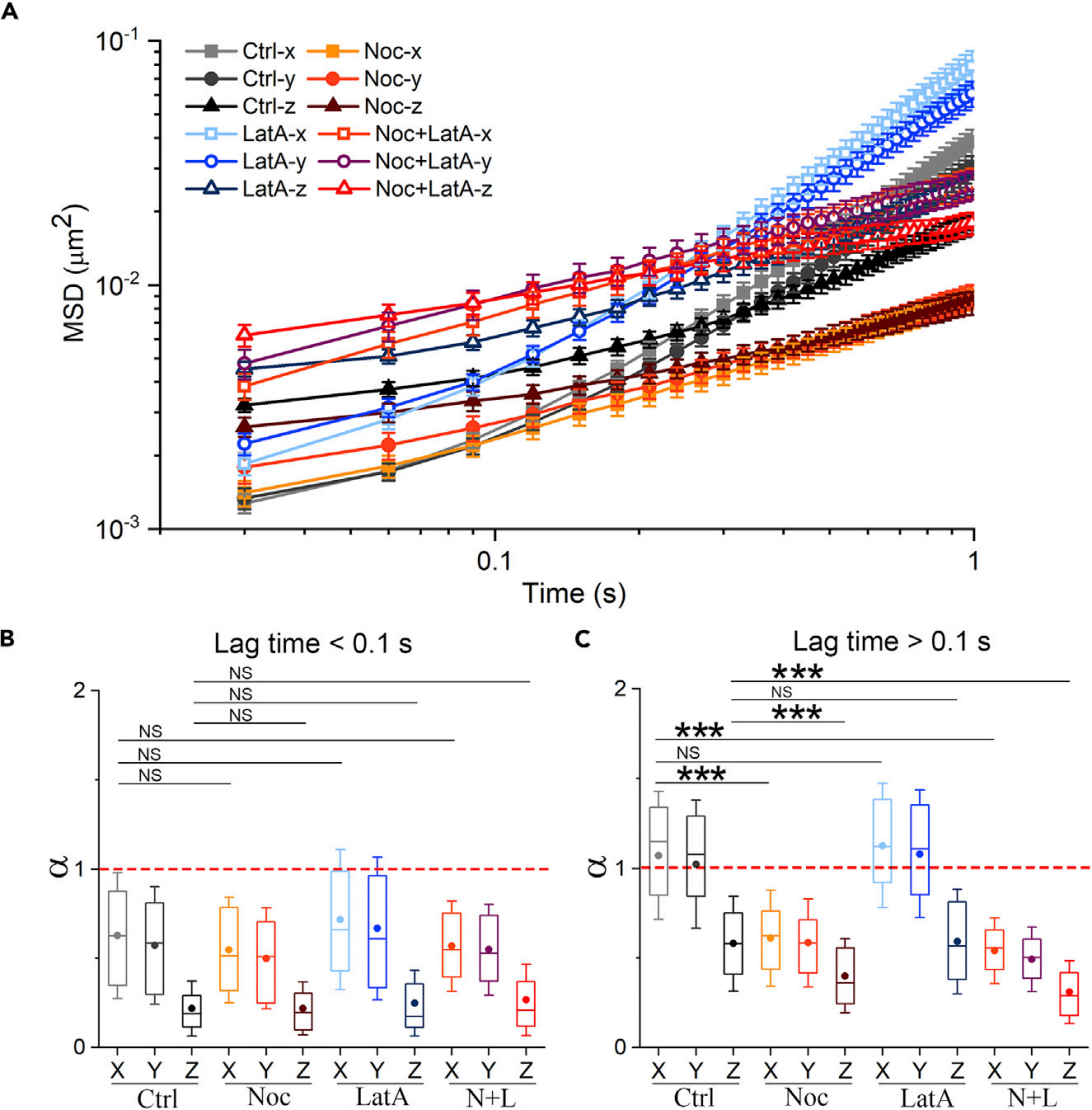
Dynamics of vesicles in the cells under different conditions (A) Averaged MSD plots in control (Ctrl, 128 trajectories in 7 cells), nocodazole-treated (Noc, 96 trajectories in 15 cells), latrunculin A-treated (LatA, 123 trajectories in 9 cells), nocodazole- and latrunculin A-treated cells (N + L, 85 trajectories in 14 cells). Error bars indicate the SEM. (B and C) Exponent α at short timescales (B) and long timescales (C). For boxplots, the line in the box means median value, the dot means mean value, box edges correspond to 25% and 75% of dataset, error bars indicate SD. ∗∗∗p < 0.001; NS, not significant.

We have shown that the long-time quasi-2D and spatially heterogeneous intracellular transport are related to the directed motions of vesicles in the lateral direction. Because microtubules and actin filaments are known to act as the paths for motor proteins that drive the directed motions and also serves as the physical constraint for the vesicles (Guan et al., 2021; Hendricks et al., 2012; Li et al., 2018b; Lombardo et al., 2019; Schuh, 2011), we next treated cells with nocodazole to depolymerize microtubules, or with latrunculin A to disrupt actin filaments (Jiang et al., 2020; Kulkarni et al., 2005). As shown by the MSD curves (Figure 3A), the two drugs differently alter the intracellular transport dynamics. Latrunculin A treatment which disrupts actin filaments has no significant influences on the exponent α at both short and long timescales (Figures 3B and 3C). Moreover, spatial heterogeneity of vesicle transport remains the same (Figure S7). However, we notice that the diffusion coefficients at long timescales have increased by about 50% after the treatment (Figure S3C), consistent with previous 2D experiments (Zajac et al., 2013). These results indicate that the actin filament has no influence on the motion type of intracellular vesicles, but only slows down the diffusion rates. Therefore, the actin filaments act more as an isotropic constraint for vesicle motions, instead of the path for directed transport.

In contrast, nocodazole treatment which disrupts the microtubules (Figure S8) reduces the 3D transport dynamics by eliminating the directed motions (Figure 3A), in agreement with previous 2D studies (Kulkarni et al., 2005; Witzel et al., 2019; Zajac et al., 2013). More importantly, we found that in the microtubule-disrupted cells, the exponent α values in three directions are greatly decreased below 1 at long timescales (Figure 3C). It suggests that the intracellular transport changes from quasi-2D motion to 3D constraint at long timescales, when removing the microtubule-based directed motion. Moreover, the heterogeneity between perinuclear and peripheral regions is also eliminated after removing the microtubules (Figure S7). When disrupting both microtubules and actin filaments, the vesicle transport is 3D constrained and laterally homogeneous, which is similar to the results with disrupted microtubules alone (Figures 3 and S8). But the diffusion coefficient at long timescales in each direction is larger in the absence of the constraint from actin filaments (Figure S3C). Together, these results demonstrate that the microtubule-based directed motion has played an important role in the temporal and spatial characteristics of intracellular vesicle transport.

### Particle diffusion on the inner membrane of vesicles

**Figure 4:**
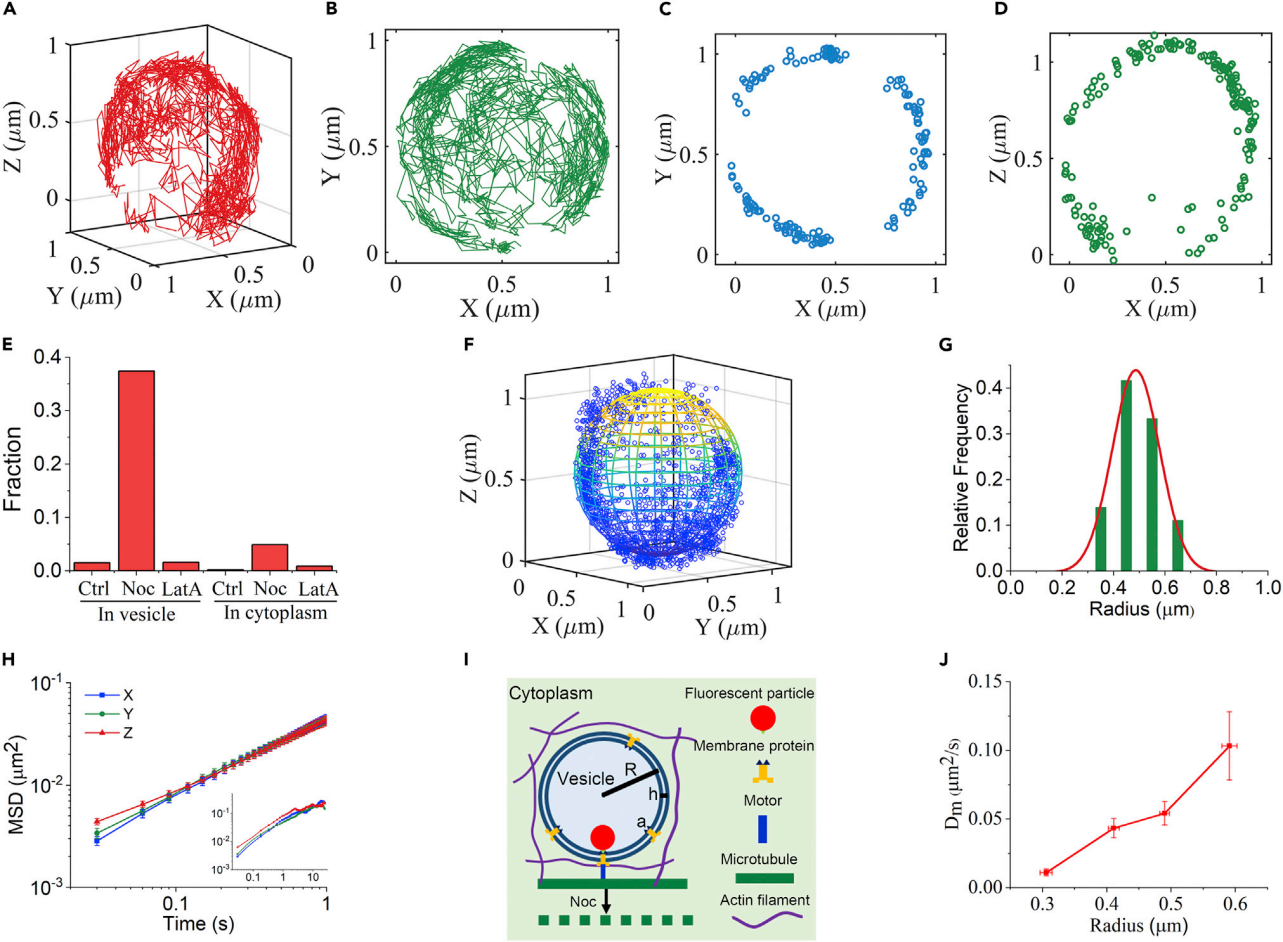
Characterization of the spherical-shell trajectories of single particles in the vesicles (A) A typical spherical-shell trajectory in 3D. (B) Projection of the 3D trajectory on the *x-y* plane. (C and D) The cross sections parallel to the *x-y* plane (C) or the *x-z* plane (D) with a thickness of 0.1 μm through the center of the sphere. (E) Fraction of spherical-shell trajectories observed under different conditions, with the particles either in vesicles or in cytoplasm. In vesicles: Ctrl, 128 trajectories in 7 cells; Noc, 195 trajectories in 16 cells; LatA, 124 trajectories in 9 cells. In cytoplasm: Ctrl, 53 trajectories in 10 cells; Noc, 102 trajectories in 10 cells; LatA, 112 trajectories in 15 cells. (F) The trajectory points are fitted to a ball with a radius of about 0.49 μm. (G) Radius distribution for 38 trajectories in 16 nocodazole-treated cells. (H) Averaged MSD curves for each direction. Inset, MSD for trajectories over 25 s. (I) Schematic plot of the motion of an endocytic particle inside a vesicle. (J) Average *D*_m_ of endocytic particles inside vesicles with different radii. Error bars indicate the SEM.

After removing microtubules, a striking phenomenon was observed that the trajectory of an endocytic particle constitutes a standard spherical shell (Figure 4A). Note that such spherical-shell trajectories can only be observed by 3D SPT, as they would be simply regarded as confined motions in the *x-y* plane by 2D SPT (Figure 4B). Cross sections of a shell trajectory in the *x-y* (Figure 4C) and *x-z* (Figure 4D) planes clearly show that almost all the dots form circular rings, and no dot appears inside the rings. Moreover, to check whether such movements inside vesicles are common for other endocytic cargos, we studied the dynamical behaviors of endocytic epidermal growth factor receptors (EGFRs) labeled by quantum dots (Li et al., 2012). Interestingly, similarly spherical-shell trajectories were found (Figure S9). These results suggest that the endocytic cargo is moving on the membrane, instead of freely diffusing within the vesicle space.

By statistical analysis of the nonstationary endocytic particles under different conditions, we found that the spherical-shell trajectories are prone to appear only after removing microtubules, but not in control or actin-disrupted cells (Figure 4E). With over more the 100 points for a trajectory, we could perform a spherical-shell fitting (Figure 4F). The spherical radius is determined to be 0.49 ± 0.11 μm in cells treated with nocodazole (Figure 4G), consistent with the lysosomes measured by transmission electron microscopy (Liu et al., 2013; Truschel et al., 2018). Then, we found that the MSD curves for each direction are perfectly overlapped (Figure 4H), consistent with the isotropic 3D motions on a spherical shell. To further examine the type of vesicles observed here, we fluorescently labeled the early endosome and lysosome, respectively, and found a good colocalization between fluorescence particles and lysosomes (Figure S10), which is consistent with previous study for endocytic particles (Rejman et al., 2004).

Next, we sought to figure out the origin of such spherical-shell movements. First, we confirmed that such trajectories are attributed to the fluorescence particles included inside endocytic vesicles, since these phenomena cannot be observed when the particles were directly loaded into cytosol (Figure 4E). Then, there are three possible motions that may lead to the appearance of spherical-shell trajectories: vesicle Brownian rotation with a fluorescence particle fixed on the inner membrane, diffusion of a fluorescence particle on the inner membrane of vesicles, or the superposition of the above two motion modes (Figure 4I). To elucidate the issue, we notice that all the scenarios are effectively equivalent to the rotational diffusion of a radial direction vector **n**(*t*), pointing from the vesicle center to the fluorescence particle. As a consequence, the mean time correlation function of **n**(*t*) can be written as $n\left(\text{t}\right)\cdot n\left(0\right)=\exp\left(-2{\text{D}}_{\text{r}}\text{t}\right)$, with *D*_r_ the effective rotational diffusion coefficient (Doi et al., 1988). Thus, the MSD of the particle on the vesicle membrane becomes

(Equation 1) $$\left\langle {\left[r\left(t\right)-r\left(0\right)\right]}^{2}\right\rangle =\left\langle R^{2}n^{2}\left(t\right)+R^{2}n^{2}\left(0\right)-2R^{2}n\left(t\right)\cdot n\left(0\right)\right\rangle =2R^{2}\left[1-\exp\left(-2D_{r}t\right)\right]\;$$

with *R* the vesicle radius.When the vesicle Brownian rotation dominates, *D*_r_ directly corresponds to the rotational diffusion coefficient of the vesicle, ${\text{D}}_{\text{r}}=\frac{{\text{k}}_{\text{B}}\text{T}}{16\text{πη}{\text{R}}^{3}}$ (for double-surfaced vesicle), with η the effective viscosity of the cytoplasm. Note that the friction contribution from the particle is negligible due to its relatively much lower value compared with the vesicle. When the second scenario dominates, *D*_r_ is related to the translational diffusion coefficient ${\text{D}}_{\text{m}}$ of the complex composed of the fluorescence particle and the linked transmembrane protein by

(Equation 2) $${\text{D}}_{\text{r}}=\frac{{\text{D}}_{\text{m}}}{{\text{R}}^{2}}\;$$

which is obtained based on the fact that in the short-time limit, the complex exhibits a quasi-2D diffusion on a local planar surface, namely $\lim_{\Delta t\to 0}{\left[r\left(\Delta t\right)-r\left(0\right)\right]}^{2}=4{\text{D}}_{\text{m}}\Delta \text{t}$. Here, the translational diffusion coefficient is related to the translational friction coefficient of the complex via the Einstein relation, $D_{\text{m}}=\frac{{\text{k}}_{\text{B}}\text{T}}{\text{γ}}$, with $\text{γ}=6\text{πη}{\text{R}}_{\text{p}}+4\text{π}\eta^{\prime }\text{h}{\left[\ln\left(\frac{\eta^{\prime }h}{\eta a}\right)-0.577\right]}^{-1}$ (Saffman and Delbruck, 1975). In the expression of γ, the first and second terms correspond respectively to the friction coefficients experienced by the fluorescence particle moving in the cytoplasm and the transmembrane protein diffusing in the phospholipid bilayer membrane, with *R*_p_ = 50 nm, a ≈2 nm, and h ≈ 4 nm separately being the particle radius, the transmembrane protein radius (Ryu et al., 2019; von Heijne, 2006), and the membrane thickness (Gan et al., 2008). Here, ${\text{η}}^{\text{′}}\;$ refers to the membrane viscosity that is about 100 times of the water viscosity (Saffman and Delbruck, 1975). And, because the water viscosity is obviously smaller than cytoplasmic viscosity η, we have ${\text{η}}^{\text{′}}\leq 100\text{η}$. Thus, for the endocytic vesicle of 0.5-micron radius, the ratio of the apparent rotational diffusion for the first scenario to that for the second scenario reads $\frac{3{\text{R}}_{\text{p}}}{8\text{R}}+\frac{{\text{η}}^{\text{′}}\text{h}}{4\text{ηR}}{\left[\ln\left(\frac{{\text{η}}^{\text{′}}\text{h}}{\text{ηa}}\right)-0.577\right]}^{-1}\leq 0.08$. In this calculation, we ignored the hydrodynamic interactions between the fluorescence particle and the inner membrane, which can enhance the friction coefficient experienced by the particle (about two times the bulk value) (Carbajal-Tinoco et al., 2007; Czajka et al., 2019). After considering this boundary hydrodynamics effect, the above ratio should be around 0.12. Therefore, it is reasonable to consider that the spherical-shell trajectories arise from the particle diffusion on the inner membrane of vesicles.

To further analyze the diffusion dynamics on the vesicle membrane, the MSD of the fluorescence particle is fitted to Equation (1), where the apparent rotational diffusion rates *D*_r_ is determined. Importantly, we found that the effective translational diffusion rate on the vesicle membranes *D*_m_ (Figure 4J), obtained from Equation (2), increases with the size of vesicles. The vesicle-size dependence of *D*_m_ could originate from the increasing deformation of the (otherwise parallel) packing structure of the phospholipid bilayer molecule with the membrane curvature, thus effectively giving rise to a more crowded medium for the diffusion of the transmembrane protein. Another possible reason is the boundary hydrodynamic effect by which as the vesicle size decreases, the inner membrane becomes more concave and thus the boundary effect in friction enhancement is more significant. Although the particle diffusion on cell membranes is extensively studied before, here, we for the first time observed the diffusion on vesicle membranes.

## Discussion

While there has been tremendous work done by 2D imaging to explore the intracellular transport that takes place in 3D cytoplasm, the problem cannot be fully elucidated without the dynamical information of axial motion. Here, by using the self-built 3D SPT microscopy, we have investigated the intracellular transport of single endocytic fluorescence particles, and revealed the anisotropic and heterogeneous characteristics of the transport dynamics, which is tightly correlated with timescales. In terms of isotropy, the vesicle transport behaves 3D constrained at short timescales (≤0.1 s) and laterally quasi-2D at long timescales (>0.1 s). In terms of homogeneity, the lateral motion of vesicles is spatially heterogeneous with the peripheral regions faster than the perinucleus. The axial motion is more homogeneous, at both short and long timescales. We further confirmed that the anisotropy and heterogeneity of vesicle transport mainly results from actively directed motion on the microtubules, which greatly increases the lateral movements in the peripheral cells at long timescales.

Our 3D results provide deep insights into the physically regulatory mechanism of vesicle transport in cells. Actin filaments confine vesicle motion, as the mesh size of actin network (around 50 nm) is smaller than the vesicles, yet it does not change the temporal-spatial characteristic of intracellular transport. In contrast, microtubules with related motor proteins enhance the vesicle motion, by generating directed motion mainly in the lateral direction. Therefore, the intracellular vesicle transport is coordinately controlled through the isotropic constraint by actin filaments and the quasi-2D directed motion along microtubules. Furthermore, because the axial motion is hardly influenced by the directed motion and shows to be more homogeneous in the cell, it is more applicable to determine the cytoplasmic moduli by measuring the axial motion of injected microbeads at short timescales, compared with the 2D passive microrheology method (Gupta and Guo, 2017; Mason and Weitz, 1995; Shen et al., 2021; Wong et al., 2004).

We further found that the cargo movements inside the vesicles are also regulated. The endocytic particles are located at the inner membrane during the intracellular transport. When disrupting the microtubules, the particles were observed to diffuse on the inner membrane and exhibit spherical-shell-like trajectories. Therefore, in addition to the roles in vesicle transport, the microtubules also control the intra-vesicle motion of endocytic cargos. These results suggest an interaction exist between the intra-vesicle cargos and the microtubules, probably through the transmembrane protein linking to both the cargo and the motor protein on microtubules (Kaplan et al., 2018). The particle diffusion on the inner side of vesicle membrane allows for accurate measuring of the vesicle size in living cells. The on-membrane diffusion rate is found to increase with the vesicle radius, which may serve as an ideal model system for investigating diffusive behaviors on membranes with different curvatures. Based on the 3D intracellular transport dynamics and its regulatory mechanism, our work may shed new light on the role of intracellular transport in cellular functions and virus infections (Ghosh et al., 2020; Zhang et al., 2021; Xu et al., 2022), and may pave the way for developing new strategies for nanoparticle-based therapeutics (Yang et al., 2021; Zhang et al., 2021).

### Limitations of the study

In this study, we studied the 3D spatiotemporal characteristics of intracellular transport, and revealed the anisotropic and heterogeneous transport dynamics. The human lung carcinoma A549 cell line was used here, which is a typically adherent cell. Further studies are needed to explore the transport behaviors in non-adherent cells as well as in 3D-cultured cells. We showed that the endocytic nanoparticles make diffusive motions on the inner membrane of vesicles when microtubules are disrupted, and suggest an underlying function of microtubules in connecting and constraining the endocytic cargos inside the vesicles. Further work would be necessary to address the physical linkage between the microtubules and the cargos inside vesicles.

## STAR★Methods

### Key resources table

**Table:** 

REAGENT or RESOURCE	SOURCE	IDENTIFIER
**Antibodies**		

Alexa-488 goat anti-mouse IgG conjugate	Thermo Fisher Scientific	A11029
Anti-EEA1- Early Endosome Marker	Abcam	ab70521
Anti-Tubulin-Microtubule Marker	Abcam	ab6161
Alexa-488 goat anti-rat IgG conjugate	Thermo Fisher Scientific	A11006

**Chemicals, peptides, and recombinant proteins**		

Dulbecco’s modified Eagle medium	Corning	10-013-CVR
FluoSpheres™ Carboxylate-Modified Microspheres	Thermo Fisher Scientific	F8803
Influx^TM^ pinocytic cell-loading reagent	Thermo Fisher Scientific	I-14402
Hoechst 33342, Trihydrochloride, Trihydrate	Thermo Fisher Scientific	H3570
Qdot™ 655 Streptavidin Conjugate	Thermo Fisher Scientific	Q10123MP
Epidermal growth factor with biotin conjugate	Thermo Fisher Scientific	E3477
Fetal bovine serum	GIBCO	10099141c
LysoTracker Red DND-99	Thermo Fisher Scientific	L7528
Nocodazole	Sigma-Aldrich	M1404
Latrunculin A	Sigma-Aldrich	L5163
Dextran T-40	BIORIGINBN	26120-10g
Penicillin-streptomycin	GIBCO	14150122
0.25% Tripsin-EDTA	GIBCO	25200-056

**Experimental models: Cell lines**		

A549	ATCC	N/A

**Software and algorithms**		

ImageJ version 1.48k	National Institutes of Health, USA	https://imagej.nih.gov/ij
MATLAB 2018a	MathWorks	https://www.mathworks.com/
Origin 2018	OriginLab	https://www.originlab.com/

### Resource availability

#### Lead contact

Further information and requests for resources should be directed to and will be fulfilled by the Lead Contact, Hui Li (huili@bnu.edu.cn).

#### Materials availability

Materials and the information used for the experiments are available upon reasonable request.

### Experimental model and subject details

#### Cell lines and culture medium

Human lung carcinoma A549 cells (ATCC) were maintained in Dulbecco’s modified Eagle medium (DMEM, Corning) with 10% fetal bovine serum (Gibco) and 1% penicillin-streptomycin (Gibco) incubated at 37 °C with 5% CO_2_. Cells were seeded in Petri dishes with glass coverslips on the bottom the day before experiments were conducted.

### Method details

#### Fluorescent particles internalization in living cells

To label the intracellular vesicles, 100-nm fluorescent particles with carboxylate coating (Molecular Probes, F8803) at a final concentration of 9 × 10^6^ microspheres/ml were added into the cell culture medium for 4 hours to be internalized into the endocytic vesicles. After that, excess fluorescent particles were removed by washing three times with phosphate buffered saline (PBS). To introduce the particles into the cytoplasm, we used a cell-loading technique based on the osmotic lysis of pinocytic vesicles (Influx-pinocytic cell-loading reagent (I-14402), Invitrogen) (Li et al., 2015). The fluorescent particles were first mixed with hypertonic solution and then added to the cells for 10 minutes. The particles are loaded into the cells via pinocytic process. After that, cells were transferred to a hypotonic medium for 2 minutes to release of the particles from the pinocytic vesicles to the cytosol. Before imaging, the cells were washed three times using PBS. To label the lysosome, 500 nM LysoTracker Red DND-99 (Molecular Probes, L7528) were added into the cell culture medium for 2 hours. To label the epidermal growth factor receptors (EGFR), cells were firstly cultured in serum-DMEM with 10 nM biotin-EGF on ice for 15 min. After being washed with cold PBS for three times, cells were incubated with 1 nM streptavidin-QDs (Invitrogen, Q10123MP) for 5 min. Then cells were washed three times with cold PBS again to remove unlabelled streptavidin-QDs. Cells were transferred to 37°C to start the internalization of EGF-QDs for at least 2 h before imaging. To prepare the dextran solution, the dextran with an average molecular weight around 40,000 was dissolved in PBS at a mass fraction of 20%. Then fluorescent particles were added to the solution at a final concentration of 5 × 10^5^ microspheres/ml.

#### Drug treatment

To disrupt the microtubules, actin filaments, or both of them, after internalization of fluorescent particles, the cells were incubated with 60 μM nocodazole, with 10 μM latrunculin A, or with both 60 μM nocodazole and 10 μM latrunculin A, respectively, for at least 30 min. The drugs were maintained in the medium throughout the experiments.

#### Microscopy

Briefly, it is built on the base of an inverted Olympus IX73 microscope, which is equipped with a 60× oil TIRF objective (1.45 N.A., Olympus) and back-illuminated EMCCD camera (DU-897, Andor Technology). A beam splitter was installed in the dual-channel simultaneous-imaging system (DV2, Photometrics) in the emission path. The splitter divides the light beam into two beams with a 30:70 intensity ratio, and each beam is sent to one-half of the EMCCD. A lens (*f* = 400 mm) is inserted into the path of the beam with 70% intensity, to produce diffraction rings. To achieve the focus-locking and prevent the vertical drift, a far-red laser (940 nm) which is totally reflected at the interface of the specimen and coverglass is continuously detected by another CCD. Any vertical drift indicated by the position shift of the far-red spot would be compensated by a piezoelectric stage. The fluorescent particles were excited by a 488-nm laser. The fluorescent particles were excited by a 488-nm laser. A CO_2_ incubation system (TOKAI HIT) was used to maintain the physiological environment of cells (37°C and 5% CO_2_). Bright field images of cells were obtained before and after the fluorescence imaging. In case of imaging fluorescent particles in dextran solution, the focal plane was selected at 20 μm above the glass surface to avoid the boundary effect.

#### 3D single-particle tracking

To determine the 3D coordinates, we analyzed the images with two steps, one step with ImageJ to get the *x*, *y* coordinates from the in-focused images, the other step with custom-written Matlab codes to get the *z* coordinates from the off-focused images. At first, specifically, the in-focused images were detected using the ImageJ plugin Particle Tracker. For each frame, individual particles were detected and localized by adjusting parameters for radius, cutoff, and percentile. The parameter of percentile was adjusted to determine which bright pixels are accepted as particles. The parameter for cutoff was set to exclude the non-particle discrimination. The parameters of linking range and displacement were adjusted to link the detected particles between frames. In our experiments, the linking range was set to 3 (no more than 3 frames). The displacement was chosen as not >2 pixels. After that, the filter option is set to 50 to only keep trajectories longer than 50 frames for further analysis. Next, the radii of the diffraction rings were determined from the off-focused images. By selecting three particle spots from an in-focused image and measuring their centers in the corresponding off-focused image, we can determine the transpose matrix between the in-focused part and the off-focused part of images. With the transpose matrix and the *x*, *y* coordinates of each particle determined from the in-focused images, we could easily define a square region of interest (ROI) containing the diffraction image of that particle in the off-focused images. The ROI is then fitted with the following equation, which is a Gaussian peak surrounded by a ring of radius *r*:

$$I=c_{0}+c_{1}\text{∗}\exp\left[-c_{2}\text{∗}\left({\left(x-x_{0}\right)}^{2}+{\left(y-y_{0}\right)}^{2}\right)\right]+c_{3}\text{∗}\exp\left[-c_{4}\text{∗}{\left({\left({\left(x-x_{0}\right)}^{2}+{\left(\text{y}-y_{0}\right)}^{2}\right)}^{\frac{1}{2}}-r\right)}^{2}\right]$$

where *I* is the intensity matrix of the ROI, *x*_0_ and *y*_0_ are the coordinates of the ring center, and *r* is the ring radius. All diffraction images were checked manually to make sure that the fitting works well. Then, to obtain the calibration between the *z* position and the ring radius, immobilized particles on coverglass were imaged, while the objective was displaced along the axis in 50 nm steps by a piezoelectric stage. By linearly fitting the piezoelectric stage displacement (*z*) with the corresponding ring radius (*r*), we obtained the calibration between *z* and *r* as *z =* −912.9 *+* 236.3∗*r*. At last, we obtained the *z* coordinates of the specific points from transforming the radius r with the calibration.

#### Immunostaining

Cells are fixed with 4% paraformaldehyde at room temperature for 20 min. Then cells are washed with PBS 3 times, permeabilized with 0.2% Triton X-100 in PBS for 10 min and blocked with 1% BSA for 1 h at room temperature. Cells are then stained by the rat anti-Tubulin monoclonal (1:500), or mouse anti-EEA1 (1:100) monoclonal overnight at 4°C. Then cells are washed with PBS 3 times and incubated with Alexa -488 goat anti-rat IgG (1:300), or Alexa-647 goat anti-mouse IgG (1:200) for 2 h. After that, cells are washed with PBS for 3 times before imaging. Nuclear are stained with Hoechst 33342.

#### Image processing

All image processing was performed using ImageJ software. The boundaries of cells were manually selected in bright-field images.

### Quantification and statistical analysis

#### Data analysis

For the reconstructed 3D trajectories with length longer than 50 frames, we calculated the global MSD using the whole trajectory with a length of 33 frames, which is about 1 s in time. The MSD curve was calculated by the equation MSD(τ) *= |r*(*t* + τ) − *r*(*t*)|^2^, where *r* is the 3D coordinates, τ is the lag time. To quantify the MSD, the exponent α is determined from the nonlinear fitting of MSD at short (*t* ≤ 0.1s) and long (*t* ≥ 0.1s) timescales respectively, with the equation MSD *= A∗t*^α^. Only at long timescales, the diffusion coefficient is further calculated from the linear fitting of MSD with the equation MSD = *2*∗*D*∗*t*, the approximation of which is acceptable as the α value is closer to 1 at the large lag times.

To identify the directed motion, the laterally 2D trajectories are used. The directional persistence of a trajectory is calculated as $<\cos\;\beta \geq \frac{1}{10\sum_{i=-5}^{5}\;\cos\;\beta_{i}}$, where β_i_ represents the change of angle between adjacent steps along the trajectory. We defined the “directed state’’ when <cosβ> is over 0.3 and α is over 1. After picking out the directed-motion segments along all trajectories, on which the consecutive points in directed stage are longer than 5 frames and their displacements are more than 1 pixel (267 nm), then we analyze the dynamic parameters of velocity, duration and run length for the directed motions in *x*, *y*, and *z* directions.

For *n* (*n* should be larger than 3) points [*x*_*i*_, *y*_*i*_ *z*_*i*_] on the surface of a 3D sphere, the residual sum of squares (RSS) follows the formula:

$$F=\sum_{i=1}^{n}{\left[{\left(x_{i}-a\right)}^{2}+{\left(y_{i}-b\right)}^{2}+{\left(z_{i}-c\right)}^{2}-R^{2}\right]}^{2}$$

where the parameters [*a*, *b*, *c*] are the 3D coordinates of the sphere center, the parameter *R* is the radius of the sphere. Then, for each parameter, we calculate the partial derivative of the RSS, and define equation equal to 0. Next, we sum the equations of all the *n* points. At last, we can calculate the values of the coordinates of the center and the radius. All the processes were done by MATLAB.

#### Statistics

For comparison, a two-tailed Student’s t-test was used. In all cases, ∗*P* < 0.05; ∗∗*P* < 0.01; ∗∗∗*P* < 0.001; NS, not significant. All the measurements were taken in three independent experiments.

## Data Availability

This study does not generate any deposited data sets. This study does not generate any deposited code. Any additional information required to reanalyze the data reported in this paper is available from the lead contact upon request.
