# Supplementary material for: Spatiotemporal three-dimensional transport dynamics of endocytic cargos and their physical regulations in cells
Source: iScience. 2022 Apr 6;25(5):104210. doi: 10.1016/j.isci.2022.104210 (PMC9035719; doi:10.1016/j.isci.2022.104210)
Supplement: Document S1. Figures S1–S10 [file mmc1.pdf]

iScience, Volume *25*

Supplemental information

Spatiotemporal three-dimensional transport dynamics of endocytic cargos and their physical regulations in cells

Chao Jiang, Mingcheng Yang, Wei Li, Shuo-Xing Dou, Peng-Ye Wang, and Hui Li

SUPPLEMENTARY FIGURES

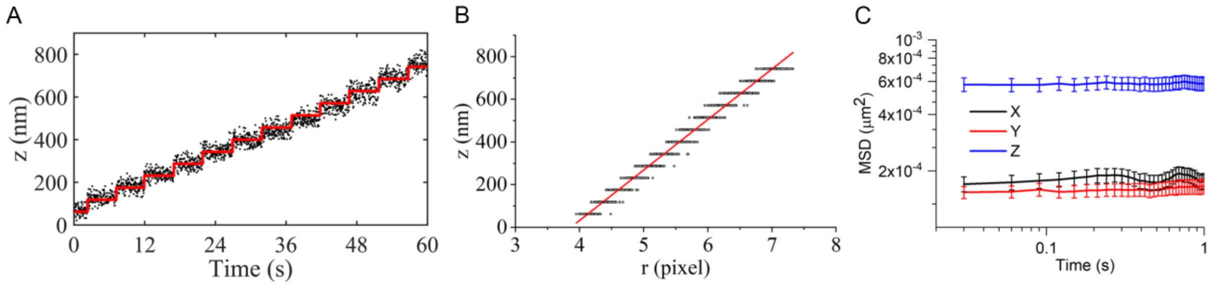

Figure S1. Calibration and resolution of the experimental setup. Related to Figure 1 and STAR Methods. (A) Sample data of the stage positions in the *z* direction (red line) and the radii of the diffraction rings (black points). (B) Calibration data between the *z* coordinate and the measured radius (*r*) of diffraction rings. The red line is a linear fit of the data, yielding $z = -911.9 + 236r$. (C) The averaged MSD curves for 15 immobilized fluorescence particles on the coverglass. The localization accuracy is determined to be lateral 10 nm and axial 17 nm.

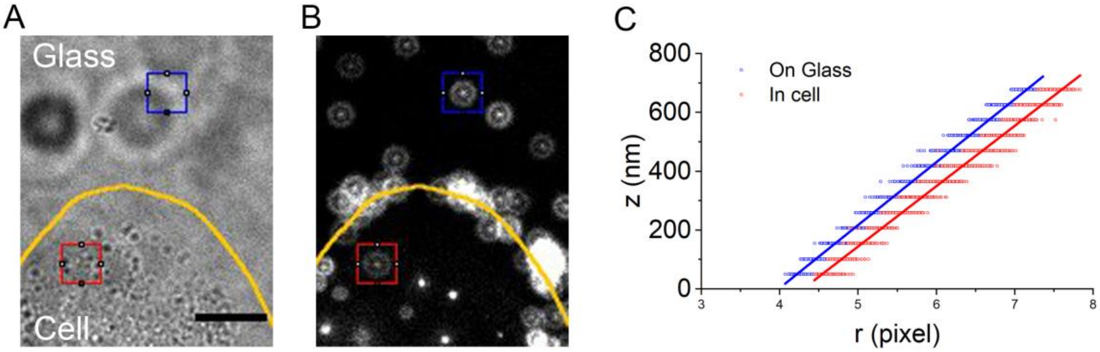

Figure S2. Comparison of the calibration curves from between the fluorescence particles in fixed cells and those on glass. Related to Figure 1. (A, B) The bright-field (A) and the fluorescent off-focused (B) image, with a particle in the fixed cell marked with a red box and a particle on the glass marked with a blue box. The cell boundary is marked in yellow. Scale bar, 10 μm. (C) Calibration data between the z coordinate and the measured radius (r) of diffraction rings. The red line is the linear fit of the data from the particle in fixed cells, yielding $z = -881.1 + 205r$. The blue line is the linear fit of the data for the particle on glass, yielding $z = -851.9 + 214r$.

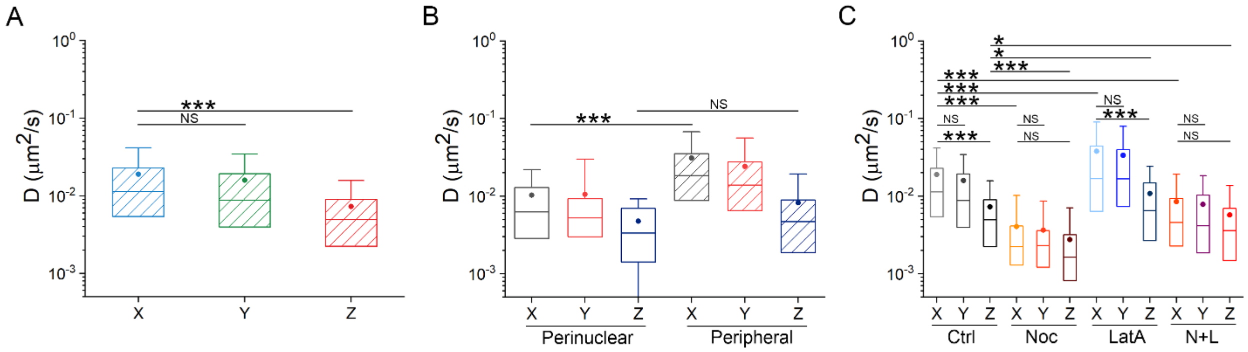

Figure S3. Results of diffusion coefficients *D* determined by the linear fitting of MSD curves at long timescales. Related to Figure 1-3. (A) Comparison of *D* at long timescales in each direction. Related to Figure 1J. (B) Comparison of *D* at long timescales in perinuclear and peripheral regions. Related to Figure 2C. (C) Comparison of *D* at long timescales under drug treatments. Related to Figure 3C.

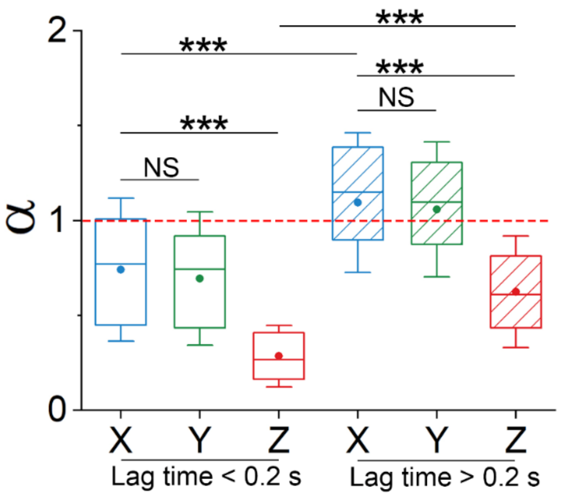

Figure S4. Exponent α in each direction at short and long timescales, with the crossover time at 0.2 s. Related to Figure 1. The results are consistent with Figure 1J with the crossover time at 0.1 s, demonstrating that the analytic results do not depend sensitively on the choice of crossover time.

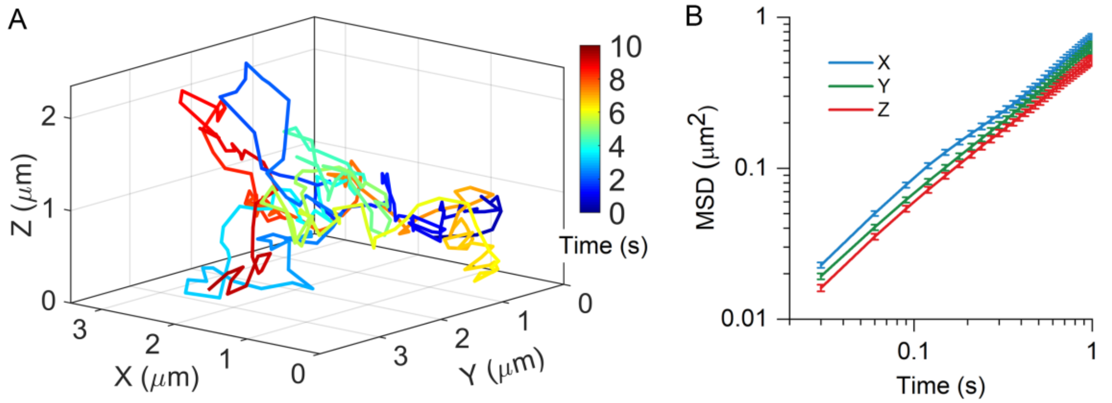

Figure S5. 3D tracking of single fluorescent particles in 20% dextran solution. Related to Figure 1. (A) A typical trajectory of the diffusing particle, with the time indicated by color. (B) MSD plots for diffusing particles (n = 38) in dextran solution in x, y and z directions. These similar MSD curves indicate the 3D isotropic diffusion.

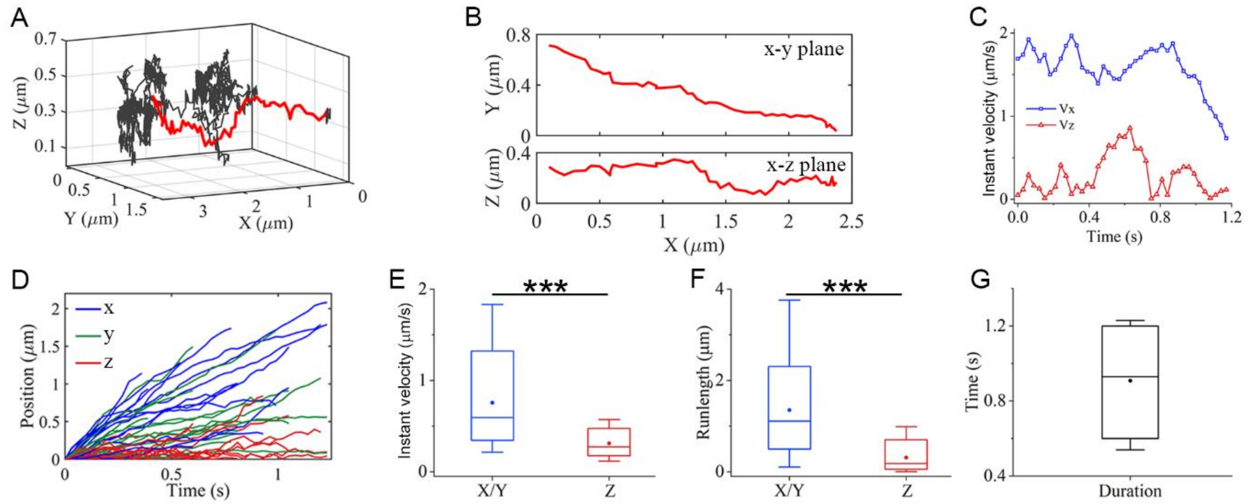

Figure S6. The analysis of directed motion in 3D. Related to Figure 1. (A) A representative super-diffusive trajectory is presented in 3D. The trajectory is segmented into directed motion (red) and diffusive motion (dark). (B) The directed motion was shown in the *x-y* and the *x-z* planes. (C) Temporal velocity of the beads in the *x* (blue) and the *z* (red) directions during the directed motion. The temporal velocity was calculated with 10 points nearby. (D) Positions in each dimension (*x*, *y* and *z*) of 15 typical directed motions were plotted with time. (E-G) The averaged velocity (E) and runlength (F) in the *x*/*y* and *z* directions, and the duration (G). 52 segments in directed motion from 18 different cells are used for statistics. ***$P < 0.001$; NS, not significant.

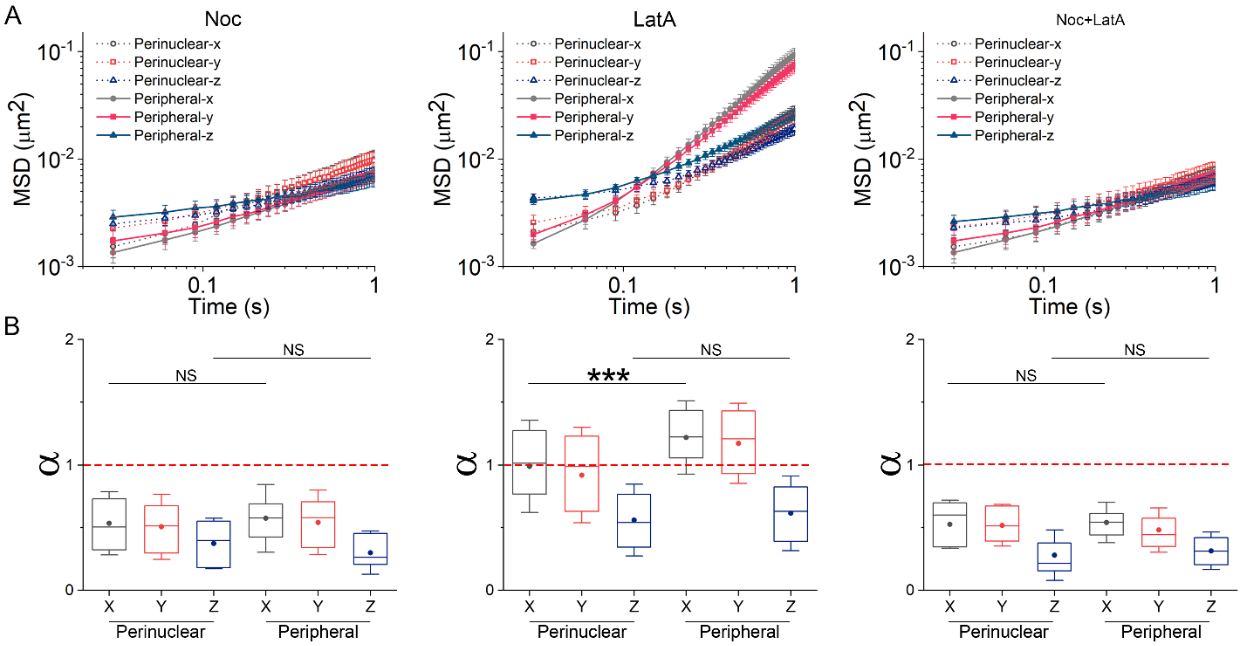

Figure S7. Spatial characteristics of vesicle transport dynamics in cells treated with nocodazole (Noc), latrunculin A (LatA), or a combination of the two drugs (Noc+LatA). Related to Figure 3. Comparison of MSD curves (A) and exponent *α* (B) determined at time-scales over 0.1 s in the three directions in perinuclear and peripheral regions. Noc, 22 (perinuclear) and 32 (peripheral) trajectories from 9 cells; LatA, 50 and 69 trajectories from 8 cells; Noc+LatA, 19 and 33 trajectories from 6 cells. ***$P < 0.001$; NS, not significant.

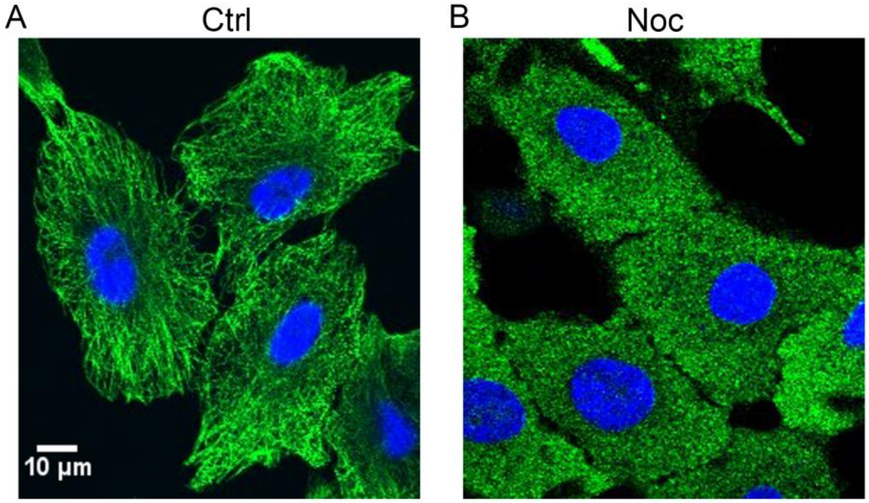

Figure S8. Confocal fluorescent images of microtubules in cells before (A) and after (B) treatment with nocodazole. Related to Figure 3. Scale bar, 10 µm.

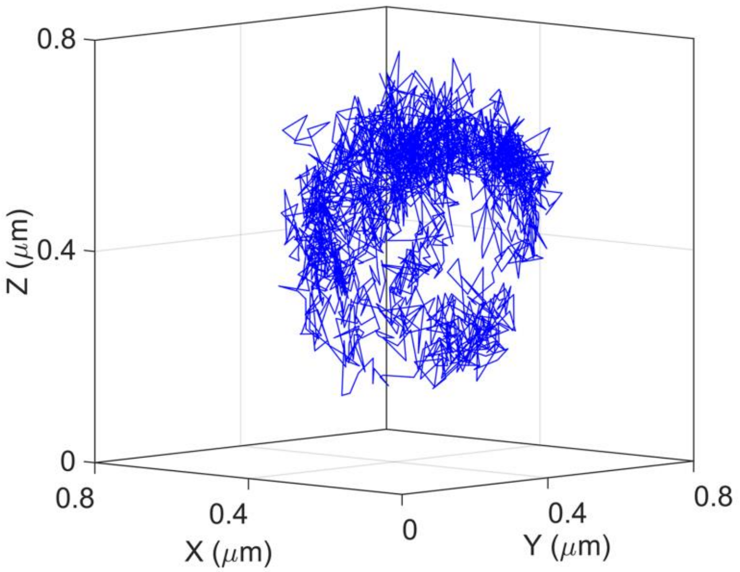

Figure S9. The 3D motion of an endocytic EGF-QD shows a typical spherical-shell trajectory, in cells when the microtubules are disrupted by nocodazole. Related to Figure 4.

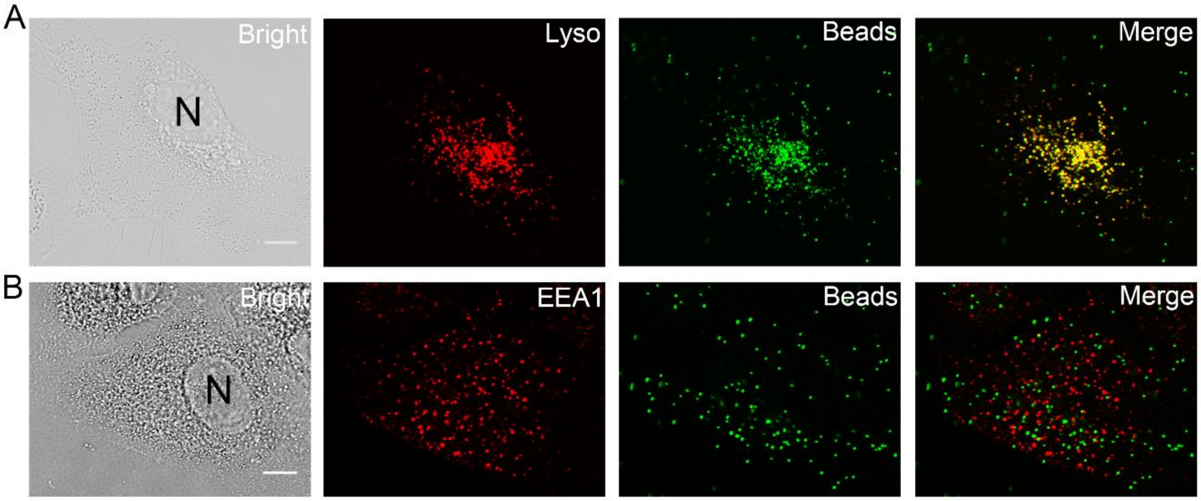

Figure S10. Co-localization experiments show the endocytic fluorescent particles are within the lysosomes after 4 h endocytosis. Related to Figure 4. (A) Intracellular location of particles (Beads) and lysosomes (Lyso). The images show the bright-field image of the cell, lysosome (red), beads (green), and the merged image of lysosomes (red) and beads (green) in the same focal plane. The yellow color indicates the colocalizations. (B) Intracellular location of beads and early endosomes (EEA1). The images show the bright-field image of the cell, early endosomes (red), beads (green), and the merged image of early endsomes (red) and beads (green) in the same focal plane. Scale bar, 10 μm.
